# A systematic review of the effectiveness of technological interventions for caregivers of people with dementia: effects on quality of life and psychoemotional variables

**DOI:** 10.3389/fpubh.2025.1579239

**Published:** 2025-05-14

**Authors:** Patricia Ferrero-Sereno, María Mendoza-Muñoz, Patricia Palomo-López, Jorge Carlos-Vivas, Patricia Luna-Castaño, Raquel Caballero-De la Calle, Miguel Ángel García-Garrido, Laura Muñoz-Bermejo

**Affiliations:** ^1^Nurse-In Research Group, University Alfonso X El Sabio, Madrid, Spain; ^2^Social Impact and Innovation in Health (InHEALTH) Research Group, University Centre of Mérida, University of Extremadura, Mérida, Spain; ^3^Physical and Health Literacy and Health-Related Quality of Life (PHYQOL) Research Group, Faculty of Sport Sciences, University of Extremadura, Cáceres, Spain; ^4^University Center of Plasencia, University of Extremadura, Badajoz, Spain; ^5^Physical Activity for Education, Performance and Health (PAEPH) Research Group, Faculty of Sport Sciences, University of Extremadura, Cáceres, Spain

**Keywords:** mental health, informal caregivers, HRQoL, stress, anxiety, depression, overload, social support

## Abstract

**Background:**

Informal caregivers of people with dementia present high levels of burden, emotional distress and low social support. Technology-based home-based interventions are presented as an alternative to providing health education, emotional support and caregiver training.

**Objective:**

To evaluate the effectiveness of technological interventions on health-related quality of life (HRQoL) and the management of psychoemotional aspects in family caregivers of people with dementia.

**Methods:**

A systematic literature review (SLR) was conducted using the Web of Science, PubMed and Scopus electronic databases, including all studies up to 7 February 2025.

**Results:**

In the review, 7,230 studies were initially identified, but only 13 met the eligibility criteria. The interventions reviewed differed in methodology when examining their impact on target variables. However, on-demand, internet- or telephone-based interventions with training activities and contact with professionals seem to improve quality of life and psychoemotional variables such as anxiety, depression and overload.

**Conclusion:**

Technological interventions using the internet and mobile applications may be useful for informal caregivers of people with dementia as they can improve quality of life and psychoemotional aspects. The interventions reviewed differed in terms of instruments and protocols when examining their impact on caregiver well-being. Therefore, more research is needed to further investigate these methodologies in order to optimize their impact and adapt them to the diverse realities of caregivers of people with dementia.

## 1 Introduction

Dementia in 2019 affected 55 million people and it is estimated that by 2050 this figure will reach 139 million due to the aging of the population (1).

Caring for a relative with dementia is a complex task with constant assistance with activities of daily living. This is compounded by associated behavioral and psychological symptoms such as agitation, erratic behavior, and loss of cognitive skills. All this generates a considerable emotional burden for caregivers, which involves significant emotional, physical and social challenges (2, 3).

The added risk for informal caregivers of dementia patients is that they face this care work without the necessary support (4). This additional burden can lead to stress, anxiety or depression (5–7). As a result of this prolonged situation, informal care at home may fail. Sometimes the level of care required is beyond the capabilities of the caregiver, who experiences a chronic stressful situation leading to an overload of care (8).

Formal interventions aim to support both people with dementia and their caregivers by addressing various support needs. These needs include acquiring relevant knowledge about dementia, obtaining information about accessible services, addressing physical and psychological health conditions and managing daily life, and maintaining social connectedness (9, 10). However, the variable availability of support services, the dynamics and complexity of family caregiving with changing support needs make it necessary to find alternative ways of providing support (11). Studies show that conventional interventions to support caregivers of older adults with dementia do not adequately meet their needs (12, 13). It has been shown that technology-based interventions can improve care for this group by relieving stress, reducing workload, optimizing care time, restoring emotional energy and improving quality of life, among other things (14, 15).

Applications developed for family caregivers of dementia patients focus on fostering constant and remote communication and monitoring of the patient (16). Interventions are now being sought that not only improve access to specialized care from home but also increase the quality of life of patients and reduce the negative impact on caregivers. In this specific situation of care for primary caregivers, digital tools have emerged as essential resources to provide individualized and remote assistance (17, 18).

The digital technologies offered for caregivers encompass various applications and platforms designed to improve the emotional and psychological well-being of caregivers (19). These solutions can provide education and training in caregiving skills as well as emotional support through telecare and mobile applications (20). These tools have been shown to provide flexibility in terms of format and timing and can reduce feelings of social isolation by facilitating communication between caregivers and health professionals (21).

Several limitations have been identified, most studies are in early stages of development, leading to interventions that focus more on technical feasibility than on assessing meaningful clinical outcomes (16). This situation limits our understanding of how these tools impact variables such as caregivers’ emotional well-being and stress. Furthermore, the lack of active involvement of caregivers and health professionals in the development and design of these types of technologies has led to the emergence of solutions that, while they may be innovative, do not always fully meet the true needs of those who ultimately use them (22, 23). On the other hand, for these technological interventions to be effective, certain methodological and practical difficulties need to be overcome. Variability in access and uptake of such technologies poses additional challenges, especially in communities where resources are scarce or among caregivers who have low levels of digital literacy (18).

In summary, while there is evidence that several of these interventions have some potential to alleviate emotional burden and promote caregiver well-being, more comprehensive and rigorous research is essential to fully understand their impactful effectiveness in depth. These studies should address not only the direct benefits for caregivers, but also the impact on the quality of life of patients suffering from dementia. This article aims to conduct a detailed review of the existing literature to assess the effectiveness of technological interventions on health-related quality of life (HRQoL) and the management of psychoemotional aspects (levels of stress and anxiety, depression, overload and social support) in family caregivers of adults with dementia. In doing so, we seek to identify current gaps in research, highlight best practices and offer recommendations for the future development of more effective and accessible interventions.

## 2 Materials and methods

In this systematic review we followed the statement Preferred Reporting Items for Systematic Reviews and Metanalysis (PRISMA) (24), which provides guidance and recommendations to the authors for the development of the research. It is a checklist to increase the transparency of the research process and the reliability of the articles published and selected in the review. PROSPERO provides the first basis for registering systematic reviews in health and, through broad consultation, promotes best practice worldwide to reduce redundancy and waste of time and resources. The research plan was therefore registered in the International Register of Prospective Systematic Reviews PROSPERO (25) (register number: CRD42025647947).

### 2.1 Literature search and selection of studies

A systematic review of the studies identified in the electronic databases was carried out PubMed, Web of Science (WoS) and Scopus up to and including 3 February 2025 in English and Spanish. PubMed, Scopus, and Web of Science (WoS) are crucial databases for health sciences research. PubMed focuses on biomedical and life sciences literature, while Scopus and WoS are multidisciplinary. WoS is a broader platform that includes several databases and allows for the analysis of scientific output and journal impact. The population, intervention, comparison, and outcome (PICO) strategy was used to structure the formulation of clinical and research questions and to guide the search and analysis of relevant references (26). The following keywords were used: “*caregiver,” “computer assisted instruction,” “computer applications,” “computer training,” “virtual classrooms,” “electronic learning,” “videotape instruction,” “virtual reality,” “online program,” “computer-based program,” “computer-based intervention,” “touchscreen,” “telephone-based intervention,” “telephone-delivered,” “app,” “application,” “health-related quality of life,” “quality of life,” “HRQoL,” “mental health,” “psycho-emotional health,” “well-being,” “stress,” “anxiety,” “depression,” “overload,” “social support.”*

The Boolean operators AND and OR have been used. They are fundamental in logic and are used to combine conditions or expressions. AND returns true only if all conditions are true, while OR returns true if at least one of the conditions is true. The logical structure of the search strategy and all the keywords, linked by Boolean Operators, that were applied in each database are presented in Table 1.

**Table 1:** Search strategy for the databases.

Data base	Search strategy
WoS	TS = (caregiv* (Topic) and “computer assisted instruction” OR “computer applications” OR “computer training” OR “virtual classrooms” OR “electronic learning” OR “videotape instruction” OR “virtual reality” OR “online program*” OR “computer-based program” OR “computer-based intervention” OR touchscreen OR “telephone-based intervention” OR telephone-delivered OR app OR application (Topic) and “health-related quality of life” OR “quality of life” OR HRQoL OR “mental health” OR “psycho-emotional health” OR well-being OR wellbeing OR stress*OR anxiety OR depression OR overload OR “social support” (Topic))
Scopus	(TITLE-ABS-KEY (caregiv*) AND TITLE-ABS-KEY (“computer assisted instruction” OR “computer applications” OR “computer training” OR “virtual classrooms” OR “electronic learning” OR “videotape instruction” OR “virtual reality” OR “online program*” OR “computer-based program” OR “computer-based intervention” OR touchscreen OR “telephone-based intervention” OR telephone-delivered OR app OR application) AND TITLE-ABS-KEY (“health-related quality of life” OR “quality of life” OR HRQoL OR “mental health” OR “psycho-emotional health” OR well-being OR wellbeing OR stress*OR anxiety OR depression OR overload OR “social support”))
PubMed	((caregiv*[Title/Abstract]) AND (“computer assisted instruction”[Title/Abstract] OR “computer applications”[Title/Abstract] OR “computer training”[Title/Abstract] OR “virtual classrooms”[Title/Abstract] OR “electronic learning”[Title/Abstract] OR “videotape instruction”[Title/Abstract] OR “virtual reality”[Title/Abstract] OR “online program*”[Title/Abstract] OR “computer-based program”[Title/Abstract] OR “computer-based intervention”[Title/Abstract] OR touchscreen[Title/Abstract] OR “telephone-based intervention”[Title/Abstract] OR telephone-delivered[Title/Abstract] OR app[Title/Abstract] OR application[Title/Abstract])) AND (“health-related quality of life”[Title/Abstract] OR “quality of life”[Title/Abstract] OR HRQoL[Title/Abstract] OR “mental health”[Title/Abstract] OR “psycho-emotional health”[Title/Abstract] OR well-being[Title/Abstract] OR wellbeing[Title/Abstract] OR stress*OR anxiety[Title/Abstract] OR depression[Title/Abstract] OR overload[Title/Abstract] OR “social support”[Title/Abstract])

### 2.2 Eligibility criteria

To be considered in the present systematic review, studies had to meet the following inclusion criteria, based on the PICOS strategy: (1) population: informal caregivers of dementia patients; (2) intervention: the intervention group must include at least one group undergoing a health education program or psychoemotional support through communication technologies; (3) comparison: must include at least one control group (CG) in which participants continue their usual activity; (4) outcomes: studies must include at least one of the following variables: HRQoL, mental or psychoemotional health (stress, anxiety, depression, overload and social support); and (5) type of study: randomized clinical trials that investigated technological interventions in caregivers of dementia patients. In addition, studies had to be written in English or Spanish and be original journal articles, thus excluding book chapters, other literature reviews, conference contributions and theses.

The choice of eligibility criteria is justified below: (1) Informal caregivers were selected because they provide daily care and face unique challenges related to caring for people with dementia. This allows the review to focus on a group that truly needs support and where interventions can have a significant impact. (2) Interventions using communication technologies are included because they represent innovative and accessible solutions for offering support, education, and resources to caregivers. This also allows for assessing how digital tools can improve their well-being and skills. (3) It is important to have a control group to compare the effects of technological interventions with those of the usual situation. This helps determine whether the observed improvements are truly attributable to the intervention and not to other factors. (4) These variables were selected because they reflect key aspects of caregivers’ well-being, such as their quality of life, levels of stress, anxiety, depression, burden, and social support. Evaluating these outcomes allows us to understand the real impact of interventions on their health and quality of life. (5) Randomized clinical trials were chosen because they are the gold standard for evaluating the effectiveness of interventions. These studies provide solid and reliable evidence on whether communication technologies truly benefit caregivers in the context of dementia.

### 2.3 Study selection

**Figure 1:**
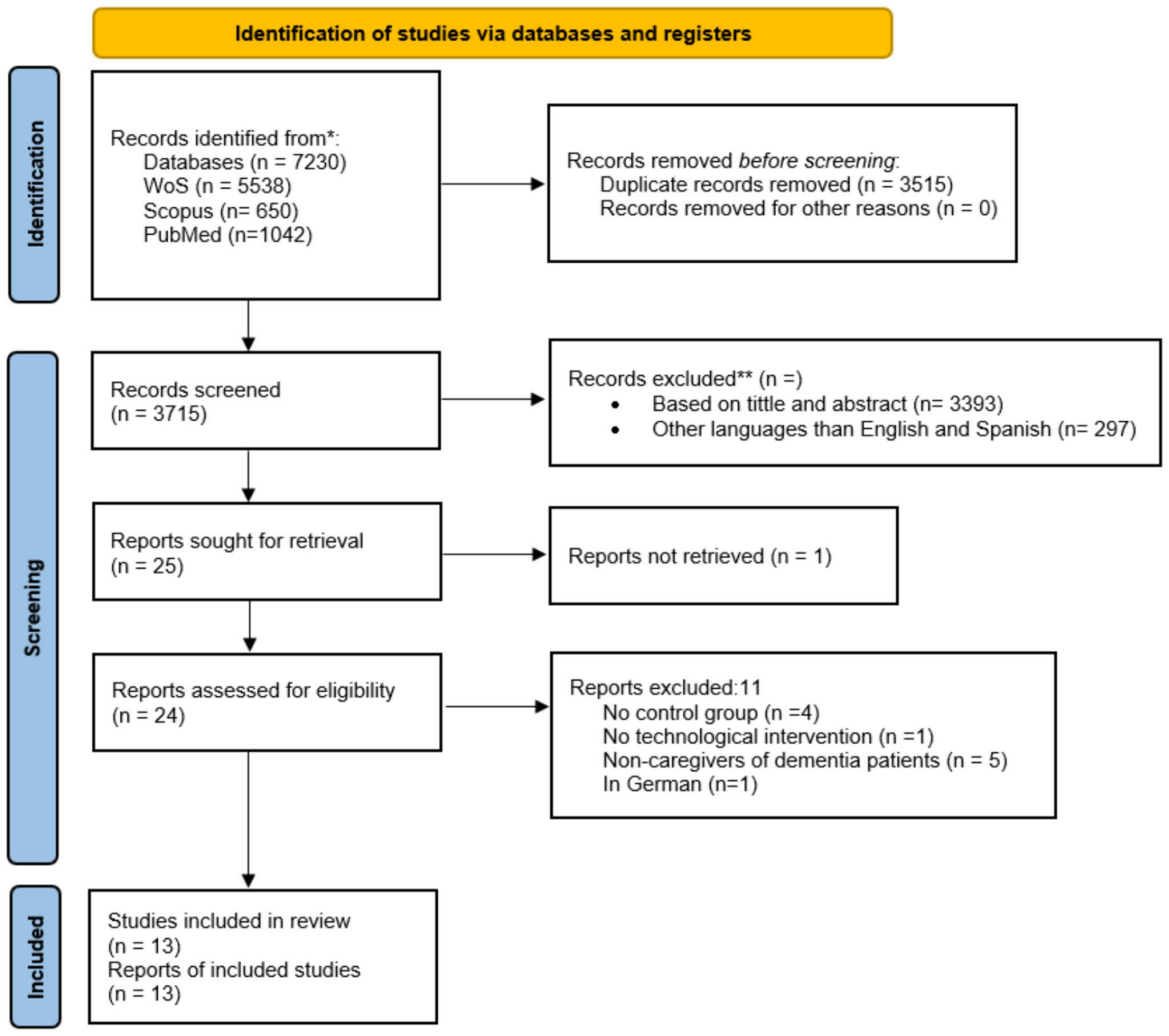
PRISMA flow diagram for study selection procedure.

The search and selection of studies was performed by two independent reviewers (PFS and LMB). They independently reviewed study titles and abstracts and excluded unrelated studies. Disagreements were resolved by discussion; if necessary, a third reviewer (MMM) was consulted to reach consensus. They then read the full articles of the remaining studies according to the eligibility criteria. The study selection process is shown in a flow chart according to the guidelines PRISMA (Figure 1).

### 2.4 Data extraction

The data extraction procedure covered the following basic information: study information (author’s name, year of publication and location), sample characteristics (sample size, age and gender), study design, intervention details (description, duration), control group and outcome measures (HRQoL, stress, anxiety, depression, overload, social support).

### 2.5 Methodological quality of studies

The risk of bias was assessed by two independent researchers (PFS and LMB) using the Physiotherapy Evidence Base scale (PEDro) (27). The PEDro scale consists of 11 items, of which only 10 (0-does not meet the criterion/1-does meet the criterion) are scored. The PEDro scale addresses randomization, allocation concealment, similarity at baseline, blinding of participants, staff and assessors, incomplete outcome data, intention-to-treat analysis, between-group comparison and measure of variability.

## 3 Results

### 3.1 Search strategy and description of studies

Initially, 7,230 studies were identified using the electronic databases mentioned above (see Figure 1). Of these, 3,515 duplicate records were identified and removed, resulting in a total of 3,715 papers. The records obtained were then sifted, excluding 3,694 manuscripts that were not related to the topic and were not written in either Spanish or English. No supplementary searches were performed in addition to the three large databases, such as checking references included in studies or other reviews, or searching for unpublished studies in trial registries. Thus, 25 articles were sought for retrieval and analysis, 1 was not retrieved and 11 did not meet the inclusion criteria.

### 3.2 Quality assessment

Thirteen studies were analyzed and scored on average more than 6 out of 10 on the PEDro Scale (Table 2). This suggests that the included studies were of moderate-high methodological quality (28).

**Table 2:** PEDro scale for methodological study quality assessment of the included studies.

Study	Items										TS	MSQ*
	2	3	4	5	6	7	8	9	10	11		
Baruah et al., (2021) (30)	1	1	1	1	0	0	1	1	1	1	8	High
Blom et al., (2015) (38)	1	1	1	1	0	0	1	1	1	1	8	High
Dichter et al., (2020) (32)	1	1	1	1	0	1	1	1	1	1	9	High
Dröes et al., (2019) (39)	0	0	1	0	0	0	0	1	1	1	4	Moderate
Lech et al., (2023) (33)	1	1	1	0	0	0	0	1	1	1	6	High
Mavandadi et al., (2017) (34)	1	0	1	0	0	0	0	1	1	1	5	Moderate
Nasreen et al., (2024) (40)	1	0	1	0	0	0	1	0	0	1	4	Moderate
Núñez-Naveira et al., (2016) (29)	1	0	1	0	0	0	1	1	1	1	6	High
Possin et al., (2019) (41)	1	1	1	0	0	1	1	1	0	1	7	High
Risch et al., (2024) (35)	1	0	1	0	0	1	0	1	1	1	6	High
Teles et al., (2022) (36)	1	0	1	0	1	1	1	1	1	1	8	High
Torkamani et al., (2014) (31)	1	0	1	0	0	0	1	1	1	1	6	High
Tremont et al., (2008) (37)	1	0	1	0	1	1	1	1	1	1	8	High

Score 0 = absent/unclear, 1 = present. TS, total score; MSQ, methodological study quality. Adapted from Maher et al. (27).

### 3.3 Characteristics of the included studies

Table 3 shows the main characteristics of the included studies. All studies involved caregivers of people with dementia aged between 44.5 and 70.0 years on average. One study did not specify the age of the caregivers (29).

**Table 3:** Characteristics of the sample.

Study	*n*	Age (media years)	Female (%)	Male (%)	Time of care	Hours of care/minimum frequency
Baruah et al., (2021) (30)	151	44.5	46.35%	53.64%	6 months	–
Blom et al., (2015) (38)	245	61.2	64.9%	35.1%	–	–
Dichter et al., (2020) (32)	38	65.8	84%	16%	6 months	4 h/day for 4 days a week
Dröes et al., (2019) (39)	119	61.8	68.07%	31.93%	–	–
Lech et al., (2023) (33)	90	68.5	71.6%	28.4%	–	
Mavandadi et al., (2017) (34)	75	70.0	97.3%	2.7%	–	4 h/day
Nasreen et al., (2024) (40)	121	51.6	69.4%	30.6%	6 months	4 h/day
Núñez-Naveira et al., (2016) (29)	61	NR	63.9%	36.1%	6 weeks	–
Possin et al., (2019) (41)	780	64.8	70.89%	29.1%	–	–
Risch et al., (2024) (35)	81	61.9	55.6%	44.4%	–	–
Teles et al., (2022) (36)	42	53.6	78.6%	21.4%	6 months	–
Torkamani et al., (2014) (31)	60	60.7 years	45%	55%	–	–
Tremont et al., (2008) (37)	60	63.3 years	NR	NR	6 months	4 h/day

Regarding the sex of caregivers, most studies recruited a significantly higher percentage of women than men. Two studies (30, 31) showed a higher percentage of male carers. Study sample numbers ranged from 38 to 90 participants in 8 studies (29, 31–37) and from 111 to 245 participants in 4 studies (30, 38–40). One study recruited 780 caregivers of people with dementia (41).

The inclusion criteria in the studies were similar in all cases and included informal caregivers of dementia patients. However, three studies included caregivers with symptoms of depression or anxiety or overload (29, 36, 38). In addition, one study (41) included caregivers of people with dementia over 45 years of age.

### 3.4 Intervention programs

As Table 4 shows, there was significant variation between studies in the number of participants in both the intervention and control groups. Thus the range of participants is very wide. There are studies with 19 participants in the intervention group and 19 in the control group (32) and studies with 512 participants in the intervention group and 268 in the control group (41). In addition, intervention programs have also varied in the instruments used. Four studies have conducted the intervention using the internet (30, 31, 36, 38), three studies conducted a telephone intervention (32, 35, 37), three studies used smartphones (29, 34, 40) and two used smartphones and the internet (39, 41). In addition, one study conducted the intervention using tablets (33).

**Table 4:** Interventions characteristics of the included studies and results of the study variables.

Study	Country	Groups	Type of intervention	Program	Duration of program and session	Frequency	A	B	C	D	E
Baruah et al., (2021) (30)	India	Intervention:74 Control: 77	Internet–based interventions	**Online skills training and support program** (dementia, caregiving, self–care, behavioral change). (dementia, caregiving, self–care, behavioral changes)	23 lessons	On demand	=	–	=	=	–
Blom et al., (2015) (38)	Netherlands	Intervention: 149 Control: 96	Internet–based interventions	**Internet Mastery over Dementia (MoD) course** (Coping; Relaxation; Organization; Cognitive Restructuring; and Communication)	8 lessons and one booster session 5–6 months	On demand	–	↓	↓	–	–
Dichter et al., (2020) (32)	Germany	Intervention: 19 Control: 19	Tele–phone intervention	**Telephone group support program** (self–care, access to care and support, communication with health care providers, communication with family and patient)	3 months	1 h every 2 weeks	=	–	–	–	=
Dröes et al., (2019) (39)	Netherlands	Intervention: 65 Control: 54	Tele–phone and internet intervention	**Individualized Support Program for Meeting Centres (iMCSP)** 3 interventions: De–menTalent (volunteering), Dementelcoach (telephone support) and STAR e–Learning (course for carers).	8 modules	NR	=	–	–	=	–
Lechet al., (2023) (33)	Germany	Intervention: 56 Control: 34	Tablet–based intervention	**DemTab application (DemTabApp)** (information on dementia and care, location of social and health care services and relaxation technique)	9 months	NR	=	–	=	=	–
Mavandadi et al., (2017) (34)	USA	Intervention: 38 Control: 37	Tele–phone intervention	**Telehealth Education Program (TEP)** (individualized care for dementia and TEP)	6 months	Minimum of 3 contacts in 3 months	–	↓	–	=	–
Nasreen et al., (2024) (40)	Malaysia	Intervention: 60 Control: 61	Tele–phone intervention (Smartphone)	**Psycho–educational intervention**	NR	NR	↑	–	–	–	–
Núñez-Naveiraet al., (2016) (29)	Denmark, Poland, Spain	Intervention: 30 Control: 31	Intervention via Smartphone or Tablet	**E–learning platform (understAID application)** (personalized support and training, and immediate response to their needs)	3 months	On demand	–	–	↓	–	–
Possin et al., (2019) (41)	USA	Intervention: 512 Control: 268	Telephone and Internet–based intervention	**The Ecosystem of Care Platform** (education, support and care co–ordination with a team of specialists in dementia)	12 months	On demand and 1 phone call per month.	–	–	↓	↓	–
Risch et al., (2024) (35)	Germany	Intervention: 41 Control: 40	Tele–phone intervention	**Telephone–based Acceptance and Commitment Therapy (tbACT)**	2 months	eight sessions/week	↑	–	↓	–	–
Teles et al., (2022) (36)	Portugal	Intervention: 21 Control: 21	Internet–based intervention	**Skills and knowledge training program for carers of people with dementia (iSupport)**	5 modules and 23 lessons	On demand	↑	↓	=	=	–
Torkamani et al., (2014) (31)	United Kingdom	Intervention: 30 Control: 30	Internet–based intervention	**Mental state and load monitoring platform (ALADDIN)** (Educational material, Contact with other caregivers, Contact with medical professionals, Remote monitoring)	9 months	2 times/day	↑	–	=	↑	–
Tremont et al., (2008) (37)	United Kingdom	Intervención:32 Control: 28	Tele–phone intervention	**Psychosocial telephone program for carers of people with dementia (FITT–D)** (emotional support, resources, self–care, and coping strategies)	12 months	weekly calls	–	–	=	↓	=

Bold type refers to intervention programme.

Differences have also been observed in the programs followed in the intervention. Online skills training programs (30), Internet Mastery over Dementia (MoD) course (38), Telephone group support program (32), Individualized Meeting Centre Support Program (iMCSP) (39), DemTab application (DemTabApp) on tablets (33), Telehealth Education Program (TEP) (34), Psychoeducational Intervention developed by smartphone (40), e-learning platform (understAID application) (29), The Ecosystem of Care Platform (41), Telephone-based Acceptance and Commitment Therapy (tbACT) (35), Skills and Knowledge Training Program for Caregivers of People with Dementia (iSupport) (36), Mental Status and Burden Monitoring Platform (ALADDIN) (31) and Telephone-based Psychosocial Program for Caregivers of People with Dementia (FITT-D) (37).

The duration and number of activities is variable, some studies have divided the activities into lessons (dementia, coping, problem-solving, organizing help, relaxation, etc.) (30, 36, 38), content modules (dementia, caregiver needs, behavioral changes, caregiving, etc.) (35, 36), components (self-care, accessing care, communication, etc.) (31, 32, 34, 39) and phases (responding to caregiver needs, assessing common problems, personalized support) (29, 41). Furthermore, the interventions analyzed varied in duration across intervention programs. Programs ranged from 2 months (35), 3 months (29, 32), 6 months (34), 9 months (31, 33) and 12 months (37, 41). However, there are interventions where it has been decided not to indicate a specific duration, but to complete the program independently without setting a specific time (30, 36, 38–40).

The variables studied were caregivers’ quality of life (30–33, 36, 39, 40), anxiety (34, 36, 38, 40), depression (29–31, 33, 35–38, 40, 41), overload (30, 31, 33, 34, 36, 37, 39–41) and perceived social support (32, 37). The stress variable was not analyzed in the selected studies and was therefore not included in Table 4.

In addition, Table 3 summarizes the variables of interest and the main results of the 13 studies analyzed for the review. Depressive symptoms together with anxiety and quality of life appear as the variables that have obtained the greatest benefit. In this sense, telephone interventions in combination with Internet-based interventions have obtained better results, and the interventions that present as an activity the promotion of knowledge about the disease, support and communication with the patient and specialists report an improvement in anxiety and depression of the caregivers (38), in depression and caregiver overload (41) and in quality of life and anxiety levels (36).

Four studies (29, 35, 38, 41) have shown significant differences between the intervention group and the control group in terms of depressive symptomatology. Three studies showed an improvement in anxious symptoms (34, 36, 38) and four improved caregiver quality of life (31, 35, 36, 40). Caregiver overload has reported significant differences in two studies (37, 41). No significant differences have been found for perceived social support in the included studies.

## 4 Discussion

The aim of this study was to review programs using technology-based interventions that have been found to be effective in improving health-related quality of life, stress, anxiety, depression, caregiver strain and perceived social support in informal caregivers of people with dementia. The findings of the systematic review indicate that digital media-based dementia interventions can improve quality of life and psychosocial status in caregivers.

Most of the interventions found were training and skills development programs, individual and group support, communication and health support (medical information, health education, psychosocial education) for informal caregivers of dementia patients. These interventions respond to the demands made by informal caregivers: the need to collect clinical information from the person they care for, medication and medical appointment reminders and follow-up of both, educational information about the disease and the treatment, courses to help caregivers and help with symptom management, contact with health professionals and sharing of information with other caregivers, and the need to provide support to caregivers (42, 43).

The studies included in this systematic review, despite their potential, show mixed results in terms of the effectiveness of the interventions. While some studies did not find statistically significant associations between certain outcomes, the direction of these results was promising for improving psycho-emotional variables and quality of life. It has been noted that on-demand interventions, where the caregiver can perform the activity without a set frequency, have had better results. In addition, more evidence of significant results was found in studies that conducted interventions based on health education, caregiver training and contact with professionals, regardless of the medium (smartphone-phone, apps or internet) used. One possible explanation could be that, although internet-based interventions or mobile apps play an important role, it is essential to recognize that face-to-face support is still needed in situations where it is required. This analysis is relevant in addressing the complexity of carers’ experiences. Consequently, the needs that carers demand and the challenges they face should be recognized in order to tailor the interventions to be developed (44).

### 4.1 Health-related quality of life (HRQoL)

One of the most studied variables in the selected manuscripts has been HRQoL. Our finding extends the existing evidence on the benefits of technological interventions in caregivers of dementia patients by showing a significant improvement in HRQoL (31, 35, 36). This finding supports the evidence that mobile and technological applications for caregivers are an effective solution to reduce their burden, improve their quality of life and avoid the negative physical and psychological consequences of caring for a dependent person (45, 46).

Technological interventions used to improve quality of life have been based on psycho-emotional care by fostering acceptance of internal and external caregiver events through a telephone intervention (35); skills and knowledge training, health education on self-care, how to provide care and how to cope with behavioral changes through an online course (36); and dementia education and contact with other caregivers and health professionals through an internet platform (31). In this sense, these studies provide information, both on the disease and the care needed, as well as physical and emotional self-care, and would meet the main need of caregivers for more information (47, 48).

However, in several articles the results have been inconclusive since they have found no differences between groups with respect to HRQoL (30, 33, 39) or, although HRQoL scores improved in the intervention groups, the differences were not significant (32, 39). For example, Dichter et al. (32) use a telephone group support program that addresses different topics such as self-care needs, access to care and support, communication with health professionals, communication with family and friends, and improving interactions with the family member with dementia. Through these group sessions, they obtain higher scores in both physical and psychological HRQoL. This could be because finding people in the same situation, as well as the new relationship created with their close environment (family and close friends), is associated with greater self-care and better emotional management, finding tools and resources to cope with the situation (49). In this sense, group interventions generate in the caregiver a feeling of psychological and social support where the participants feel that they share the same burden and situations, and for this reason could increase HRQoL scores.

### 4.2 Anxiety and depression

Anxiety and depression are indicators of emotional distress (50, 51). The use of Internet applications is an effective way to provide interventions to support family caregivers of people with dementia throughout the caregiving process (52). In this regard, our findings show a reduction in symptoms of anxiety (34, 36, 38) and depression (29, 35, 38, 41), which would support that technological interventions for caregivers have a positive effect on mental health.

Other studies have found improvements in anxiety and depression scores in caregivers, although they were not significant (31, 36, 37). In these cases, perhaps a longer period of use of the platforms or mobile applications may be necessary to see if it is effective and significant in the long term (53).

Interventions that have demonstrated an improvement in anxiety symptoms have included an Internet-based course, which included coping, relaxation, organizing help to others, cognitive restructuring and communication (38); a Telehealth Education Program, which included health and psychosocial education and addressed topics such as communication skills, behavioral and stress management and coping skills (34); and a Skills and Knowledge Training Program for caregivers of people with dementia (36). Studies that add specific functions to meet the emotional needs of informal caregivers are scarce, however, it has been shown that programs that include aspects related to caregiver mental health report an improvement in symptomatology (42).

Interventions that have achieved a reduction in depressive symptoms have used a Smartphone or Tablet platform that informs, supports and trains informal caregivers in a personalized way, providing immediate response to their needs (29); a telephone and internet-based supportive care intervention that provides education, support and care coordination with a team of dementia specialists (41); and telephone intervention that includes therapy for acceptance of aversive internal and external events, choice of meaningful courses of action consistent with personal values, and value-driven action (35). In all of them, personalized attention is provided to meet the individual needs of caregivers. In this regard, coaching and telecoaching have been shown to increase caregivers’ feelings of competence, reduce psychosomatic complaints, decrease depression in caregivers, and improve self-efficacy (54, 55). In addition, it has been suggested that ICTs are a suitable tool to teach caregivers better ways to cope with caregiving stress (56).

### 4.3 Caregiver overload

Psychosocial interventions focused on changing perceptions, timely information (psychoeducation), improving coping skills, and encouraging the use of care and support services can help people with dementia maintain a good quality of life and prevent informal caregivers from becoming overburdened (39). However, most studies that have assessed caregiver overburden have either found no differences between groups (33, 34, 39) or the burden has improved but the changes have not been significant (30, 36). This could be because in addition to participating in the study, caregivers must continue to provide care to a person with an evolving disease, so caregiving tasks do not decrease (57).

Three studies have found an improvement in caregiver burden scores after intervention. Two interventions are based on tele-phonic care for psychosocial support (37, 41) and an intervention is internet-based with educational material, contact with professionals and remote monitoring (31). In this sense, it has been shown that social support is a variable that positively influences caregiver burden (58).

### 4.4 Perceived social support

Internet-based interventions have been explored as a means of extending training and support to caregivers of people with dementia, either as a complement or as an alternative to usual care (59). Among the technological alternatives that have been analyzed in this review, two studies have evaluated the perceived social support after the intervention (32, 37). No differences were found between groups; however, some improvement in perceived social support was found in the results of Tremont et al. (37), an intervention that included emotional support sessions, information on health resources and coping training. Although some references have been found where support for caregivers through training and education programs, support groups, counseling, and web-based and multicomponent interventions have been shown to be moderately effective in improving perceived support and caregiver competence (60), in this case the results have not shown an improvement in perceived social support. This could be due to the lack of technological interventions focused on connecting families to community support systems or to the local community of long-term services and supports (such as home care agencies, voluntary respite or adult day care programs, nursing homes, etc.) or because support programs are delivered on an *ad hoc* basis during the day and the caregiver must continue to cope with the situation at home on an ongoing basis. This fact may require future interventions to incorporate more technological peer-to-peer support that directly connects caregivers to long-term services and support to address this gap and potentially improve feelings of social support in this population.

Considering the analysis of the studies, internet- or telephone-based programs are valuable and worthy of consideration for caregivers of people with dementia, at least as a complement to usual care or to new interventions, as they are low-risk and show signs of effectiveness in key outcomes. Furthermore, it should be noted that caregiver engagement and adherence to digital interventions only work if caregivers actually use them. Therefore, mentioning adherence and dropout rates would be interesting, or successful recruitment and retention methods that have been shown to keep caregivers using technology-based interventions would be useful to the field. Mentioning the accessibility of digital interventions for this population, particularly the use of user-friendly digital interfaces, technical support, or technology training protocols, or analyzing how the studies consider the needs of diverse caregiver populations who use technology (such as rural caregivers, older caregivers, and those with low technical literacy), would demonstrate a more comprehensive understanding of the research conducted and help identify better ways to implement technology-based strategies for caregivers in practice.

Furthermore, we should mention that in this field, artificial intelligence could play a key role in interventions for caregivers of people with dementia in the near future. It is even beginning to be used to offer support and resources that facilitate caregiving, such as platforms that provide personalized information, medication or appointment reminders, and tools that help monitor the well-being of people with dementia. Therefore, it would also be interesting to introduce this type of intervention in future studies. This review covers the most recent studies published up to 2025, ensuring that the information collected is current and reflects the latest advances in the field. Furthermore, it has specifically focused on specific and highly relevant interventions, such as randomized controlled trials (RCTs) and other types of intervention trials, as these studies provide high-quality evidence on the effectiveness and safety of implemented strategies. Choosing to focus on these types of studies allows for more robust and reliable conclusions about how communication technologies can be used to support informal caregivers of people with dementia, ensuring that the results are relevant and applicable to clinical practice and healthcare decision-making.

### 4.5 Limitations and future directions

This review has limitations that need to be considered. Firstly, there is a high heterogeneity of interventions, content and instruments, which makes it difficult to make comparisons across studies and a more robust and accurate analysis. Secondly, the heterogeneity in sample sizes and the lack of statistical association in some results pose challenges for a more comprehensive interpretation. Thirdly, there were studies whose inclusion criteria required participants to be overburdened, which can lead to bias in the results. Fourth, the search was restricted to RCTs and intervention-based studies. We did not include potentially illuminating qualitative or mixed methods studies on caregivers’ use of technology that might shed light on why certain interventions failed or which components of technology caregivers find most useful. Finally, in many studies, validation of the application or technological platform is carried out and the evaluation of the intervention is a pilot project, and the long-term effects of these interventions are unknown. In view of this, the need for future mobile or internet-based interventions using standardized protocols and approaches to achieve comparable results is highlighted. It would be of interest to include longitudinal studies involving technology platforms or applications in order to assess the effect of interventions over time on psychoemotional and quality of life variables. Likewise, including the measurement of the caregiver stress variable could be a future line of research. It would also be interesting to know the effects of the interventions by gender, since it is women who carry out family care tasks more frequently.

## 5 Conclusion

In general, interventions aimed at caregivers of people with dementia show promising results in terms of improving psycho-emotional variables (such as anxiety and depression) and caregivers’ quality of life. Technologies, such as internet-based programs, mobile applications or phones, seem to have a positive impact, although results vary in terms of their statistical significance.

The results obtained suggest that interventions aimed at caregivers have a positive impact on their psychoemotional well-being, with significant variability in the methodologies employed. It has been pointed out that the interventions on demand, which allow the caregiver to develop the activities in a flexible way, have shown better results, possibly due to the autonomy they provide to the caregiver. Likewise, studies that have integrated health education components, specific training of caregivers and contact with professionals have achieved significant results in terms of improved emotional well-being. These findings underline the importance of adopting personalized and accessible approaches that include education and ongoing support, tailored to the needs and preferences of caregivers. However, further rigorous research that explores these methodologies in more depth is essential to optimize their impact and adapt them to the diverse realities of caregivers of people with dementia.

## Data Availability

The original contributions presented in the study are included in the article/supplementary material, further inquiries can be directed to the corresponding authors.
